# Development of Mathematical Models Evaluating Presence of Coronary Calcification Independent of Computed Tomography (DEPICT): Radiation-Free Evaluation of Coronary Atherosclerosis

**DOI:** 10.31083/RCM46777

**Published:** 2026-06-15

**Authors:** Yinze Ji, Aimin Dang, Naqiang Lv

**Affiliations:** ^1^Premium Care Center, Department of Cardiology, Fuwai Hospital, Chinese Academy of Medical Sciences & Peking Union Medical College, National Clinical Research Center for Cardiovascular Diseases, National Center for Cardiovascular Diseases, 100037 Beijing, China; ^2^School of Public Health and Emergency Management, School of Medicine, Southern University of Science and Technology, 518055 Shenzhen, Guangdong, China

**Keywords:** coronary artery calcification, radiation-free evaluation of arterial calcification, machine learning, precision medicine, prediction model

## Abstract

**Background:**

The dependence of the acquisition of the coronary artery calcification score (CACS) on computed tomography (CT) has drawbacks, including the ethical concerns of radiation exposure in the care of patients with non-cardiovascular diseases, where CACS has been shown to correlate with its prognosis. Significant heterogeneities exist between patients with and without coronary artery calcification (CAC). Mathematical formulae using medical history and common, non-invasive test results enable cheap, ready assessment of CAC and subsequent research into how it can be used for clinical decision making.

**Methods:**

694 patient records of visits to Fuwai Hospital, Chinese Academy of Medical Sciences and Peking Union Medical College from 2009 to 2023 were partitioned into a training (visited before 2023) and an independent validation set (visited in 2023). With age, gender, current smoking, diabetes, low-density lipoprotein cholesterol (LDL-C), reduced renal function, usage of statins and aspirin as candidate predictors, five logistic regression models were built under two paradigms. Bootstrap resampling was employed for internal validation, followed by external validation and calibration on the validation set. Models built under each paradigm were compared, followed by head-to-head comparison of the “best” models built under each paradigm with a comprehensive criteria involving both model performance and predictor parsimony.

**Results:**

694 records were used for modeling, with 536 and 158 records in the training and validation set respectively. Model 1 (*c* statistic upon external validation: 0.77) outperformed other models built under Paradigm 1 while Models 4 (*c* statistic upon external validation: 0.79) and 5 (*c* statistic upon external validation: 0.79) built under Paradigm 2 outperformed Model 1. Model 5 was more parsimonious in predictors. All models were well calibrated.

**Conclusion:**

With gender, current smoking, LDL-C, age, diabetes and reduced renal function as predictors, Model 5 outperformed other models and was hence recommended for further use. By assessing the presence of CAC with medical history and blood test results instead of CT, our model offers an approach to immediate, radiation-free assessment of CAC, which may further unleash the clinical utility of CAC in clinical practice that may have remained unraveled.

## 1. Introduction

Coronary artery calcification (CAC) is associated with elevated cardiovascular risk. Research has revealed the crucial role the coronary artery calcification score (CACS) plays in the diagnosis of early, subclinical coronary artery disease [1]; risk stratification of diabetic [2], hypertensive [3], elderly populations [4] and smokers [5]. CAC is not only associated with coronary in-stent restenosis and in-stent thrombosis, conditions that are both associated with stent under-expansion [6], but also associated with prognosis of certain non-cardiovascular diseases (CVDs) (e.g., carcinomas, hip fracture, chronic obstructive pulmonary disease) [7].

Significant heterogeneities exist between those with CACS = 0 and those with CACS >0. Patients afflicted by CAC suffer from an increased risk of adverse events, regardless of its severity [8]. A CACS of 0 is also an indicator of very low 10-year mortality in both middle-aged, elderly [9] and younger patients [10], a marker of good prognosis in those with a huge risk factor burden [11], lipid profile impairment [12] and metabolic syndrome [13] and the strongest protective factor among several protective factors [14]. Statin use was associated with a reduction in risk of major adverse cardiovascular events (MACEs) in patients with CACS >0, while those with CACS = 0 did not enjoy such benefit [15]. These results showcase the disparities between the two populations and the need for distinguishing them with methods including, but (as this study presents) not confined to, computed tomography (CT) exams.

Despite the clinical significances found by medical literature, clinical utilities of CAC remain underdeveloped, with total CACS being extensively researched but playing a limited role in clinical practice. Inability to repetitively acquire CACS in the same simple way doctors acquire estimated glomerular filtration rate (eGFR) is one of the reasons. In practice, CACS may be routinely reported when coronary computed tomography angiography (CCTA) is done, limiting the number of patients with more than one CACS result each year. However, repetitively acquiring the “true” CACS values with CT- a modality with high sensitivity, specificity, less inter-observer variability- leads to concerns of over-diagnosis, radiation exposure and medical costs associated with repetitive CT scans, especially in patients with non-CVDs (e.g., hip fracture). The promising utility of CAC in the future when more of them are exploited hardly justify the costs patients pay now for CT exams if quantitatively ascertaining CAC is the sole purpose. More specifically, despite the presence of multiple risk prediction models [16,17], including ones where CACS has been demonstrated to significantly enhance its discriminative performance [16], its application in clinical practice has been regretfully confined due to the implausibility of universal CAC testing, a phenomenon recognized in a lately published editorial on *Journal of the American Medical Association* [18]. In fact, as has been pointed out in the editorial [18], as much as most individuals benefit from CAC testing, asymptomatic individuals in the United States do not receive payment from Centers for Medicare & Medicaid Services or, in many cases, private insurance. Therefore, $ 50 to $ 400 has to be paid for this examination without reimbursement [18]. Self-referral has increased the availability of CAC testing [18], but certainly not to the extent of blood lipid and glucose testing due to issues such as radiation exposure. Therefore, a model enabling radiation-free assessment of CAC can partially fulfil the need of asymptomatic individuals in the sense of informing them whether they are likely to be affected by CAC or not.

Given (1) the need to further unleash the clinical utility of CAC, (2) the challenges faced when attempting to ascertain CACS by CT, (3) the heterogeneities of populations with and without CAC and (4) prior success of addressing the inconvenience of determining glomerular filtration rate by a mathematical formula, the Development of Mathematical Models Evaluating Presence of Coronary Calcification Independent of Computed Tomography (DEPICT) study sought to address this problem by developing mathematical models. More specifically, models were developed to evaluate the presence of CAC to enable cheap, radiation-free and readily available assessment of the presence of CAC via medical history and common, non-invasive test results for clinical practitioners and patients.

## 2. Materials and Methods

### 2.1 Data Sources and Study Population

Data from 1035 hospitalizations and outpatient visits of Fuwai Hospital, National Center for Cardiovascular Diseases, Chinese Academy of Medical Sciences and Peking Union Medical College, a teaching tertiary hospital, from September 2001 to August 2023 were retrospectively collected. Inclusion criteria of the DEPICT study were: (1) Age ≥18 years; (2) No previous history of percutaneous coronary intervention (PCI), coronary artery bypass graft (CABG) or heart transplantation; (3) Underwent multidetector row helical computed tomography (MDCT) exams that gave rise to reports of CACS values and laboratory tests, with a time lag between them no longer than one month. Exclusion criteria were established diagnosis of autoimmune diseases or familial hypercholesterolemia. Based upon these criteria, data from 694 records from September 2009 to August 2023 eventually entered the modeling process, with 536 records of patients who visited Fuwai Hospital from 2009 to 2022 in the training set, and an independent cohort (i.e., none of the patients therein had any record in the training set) of 158 patients who visited Fuwai Hospital in 2023 in the validation set.

### 2.2 Data Collection

Patient electronic medical record data, including information on age, gender, diagnosis, history of operations, use of medications prior to laboratory exams, smoking and alcohol drinking status, family history, laboratory test and CACS results were collected. The DEPICT study issued no request for additional data collection as all data analyzed were generated by doctors’ comprehensive plan of diagnosis and treatment within standard of care.

### 2.3 Statistical Analysis & Mathematical Modeling

All modeling processes were carried out using Statistical Analysis System (SAS) Version 9.4 TS1M5 (SAS Institute Inc., Cary, NC, USA). The DEPICT study adhered to the Transparent reporting of a multivariable prediction model for individual prognosis or diagnosis (TRIPOD) guidelines [19].

#### 2.3.1 Univariable Analyses

Patients were divided into two groups: total CACS >0 group and total CACS = 0 group. χ^2^ tests were performed for intergroup comparisons of categorical variables. For continuous variables, *t *tests or Wilcoxon sum-of-rank tests were performed (chosen as appropriate) based on the normality of each group, which was ascertained by *p *values of Shapiro-Wilk test or Kolmogorov-Smirnov test (chosen as appropriate), histograms, percentile-percentile plots and quantile-quantile plots. Generalized additive models were built to explore nonlinear associations with the logit transform of the probability that the patient’s CACS was larger than 0 (logit*p*). Thin-plate regression splines were chosen as smoothers because of data-driven degrees of freedom of spline terms, availability of fit statistics and the resulting ready comparison of models if adjustments had been made to the number and location of spline knots.

#### 2.3.2 Predictor Specification

Given the sample size of the DEPICT study, there was a possibility of overfitting and occurrence of testimation bias [20] when *p*-value-based variable selection was used in modeling. To circumvent this, we specified candidate predictors in the model based on subject matter knowledge, accessibility in clinical practice and results of univariable analyses. *p*-value-based variable selection was not performed. Predictors specified were: gender, age, current smoking status, diabetes, serum low-density lipoprotein cholesterol (LDL-C), ratio of serum LDL-C and serum high-density lipoprotein cholesterol (HDL-C), eGFR (calculated by Chronic Kidney Disease epidemiology collaboration (CKD-EPI) creatinine equation) and usage of medications (pre-laboratory test usage of aspirin and statins). Of note, serum LDL-C and eGFR referred to the baseline (first available result after admission) levels if CT were conducted during hospitalization and levels of the most recent laboratory test as compared to the time of conducting CT if the latter were conducted in the outpatient clinic or emergency department. Medication use refers to use of aspirin and statins in the 7 days preceding laboratory tests. Readers and potential users of our model should be reminded that the time lag of laboratory tests and CT was set to be less than 1 month in the patient selection criteria mentioned above. The assessment of each and every predictor was blinded for the outcome (presence of CAC) and other predictors. Details regarding the considerations of specification of these variables are given in **Supplementary Material Ⅰ**.

#### 2.3.3 Outcome Specification

The outcome assessed was presence of CAC, which was defined as total CACS >0. More specifically, CACS referred to Agatston CACS calculated from MDCT scans. The calculation process was blinded.

#### 2.3.4 Selection of Modeling Method

Among the vast number of methods potentially suitable, we chose logistic regression, whose superiority or non-inferiority in predictive modeling, especially in the medical field where datasets comparable to certain datasets in other fields (e.g., the Modified National Institute of Standards and Technology (MNIST) database) in size is hardly available. The StatLog project [21], a systematic comparison of statistical modeling methods of binary outcome, found the advantages in predictive performance of neural networks and Classification and Regression Trees (CARTs) over logistic regression existent only in larger data sets. Ennis et al. [22] found that in general, generalized additive models, CARTs, and multivariate additive regression splines (MARS) did not outperform logistic regression in both *c*-statistic and log-likelihood in a medical setting. None of the predictive performances of various variants of neural networks and various methods preventing overfitting that they examined were better than the logistic regression model. No improvement in predictive performance of traditional and modern tree-based methods was observed as compared with logistic regression in large (sample size larger than 15,000) datasets [23,24]. The “white box” nature of logistic regression carries ready and clear interpretation of its results, which is another advantage over alternatives including machine learning methods.

Logistic regression is a versatile modeling method that is capable of calculating the probability that an individual not involved in this study has CAC via the inverse of logit transform (e.g., $p=\frac{\exp\left(\beta_{0}+\beta_{1}*\text{ Gender }+\beta_{2}*LDL-C+\beta_{3}eGFR\right)}{1+\exp\left(\beta_{0}+\beta_{1}*\text{ Gender }+\beta_{2}*LDL-C+\beta_{3}eGFR\right)}$ , where p stands for the individual’s probability of having CAC, LDL-C stands for level of low-density lipoprotein cholesterol, eGFR stands for level of glomerular filtration rate, $\beta_{0}$ , $\beta_{1}$ , $\beta_{2}$ and $\beta_{3}$ are coefficients that can be calculated from the data). Based on the data at hand, the DEPICT study attempts to calculate the optimal cut-off probability above which the patient is assessed to have CAC, which can subsequently be compared with the probability calculated for the new individual, resulting in an assessment of whether the individual has CAC.

#### 2.3.5 Development of Mathematical Models

Prior to the modeling process, the entire dataset was partitioned into the training (536 records) and testing dataset (158 records). The entire model-building process was exclusively conducted on the training set. With biological plausibility and model generalizability in mind, two modeling paradigms (Paradigm 1: dichotomization of continuous predictors + inclusion of medication history + possible inclusion of interaction vs. Paradigm 2: no dichotomization or inclusion of medication history + no inclusion of interaction) with multiple strategy variants were adopted, starting from simple, parsimonious models to more complex ones with two-way interaction terms with all of the continuous predictors dichotomized, a method in line with the usual practice in logistic regression modeling. We next cancelled predictor categorization of continuous predictors as long as the coefficients of the model generated were biologically plausible. No nonlinear term was included in any of the models. Collinearity and its associated ill-conditioning caused by the weighted matrix of predictors were examined by diagnostic statistics computed under both intercept-considered and intercept-not-considered conditions. Rescaling of predictors [25] was conducted to remove collinearity and its associated ill-conditioning if they had been indicated by diagnostic statistics when the intercept was considered but not indicated when the intercept was not considered. Optimal cut-off posterior probability of each model was searched among a grid of probabilities with an interval of 0.01. The posterior probability corresponding to the largest Youden index was chosen as the optimal cut-off. Complete case (CC) analysis revealed collinearity and ill-conditioning of the Fisher information matrix it causes in some of the models, presenting challenge to missing data treatment. Methodological research studies are still warranted for data imputation methods, including the combination of model internal validation via bootstrap resampling and data imputation [20]. Collinearity and ill-conditioning of the information matrix complicate the problem, for which, to date, no guideline has been retrieved. Therefore, we conducted CC analyses in the modeling and validation processes. Patterns of missing data and numbers as well as proportions of records with each missing data pattern in the modeling process of each model are given in **Supplementary Material Ⅰ**.

#### 2.3.6 Model Validation

2.3.6.1 Internal Validation

Bootstrap resampling was used for model internal validation as recommended [26]. In short, 2000 balanced bootstrap samples were generated from the training set, followed by training on each of the bootstrap samples and testing on the original training set. Optimism was calculated by averaging the difference in the performance statistics (e.g., *c *statistic) between those of the model generated by each of the bootstrap samples and those of the very model generated by the bootstrap samples on the original sample. Optimism of performance statistics was subtracted from the corresponding performance statistics of the model to be internally validated, generating optimism-adjusted performance.

2.3.6.2 External (Temporal) Validation

Temporal validation has been documented as a valid approach in external validation [20]. Following the suggestion of Steyerberg [20], data from the visits in 2023 were saved for external validation to test the performance of models on more recently visited patients, which is more clinically relevant. All models built were validated on the independent validation set, generating discriminatory statistics. Calibration of models was only performed on the validation set, as calibration on the training set and during the internal validation process provided limited information [20]. Calibration plots were drawn with confidence bands of calibration curves while statistics of calibration in-the-large, weak calibration, calibration intercept, calibration slope and their 95% confidence intervals (CIs) and *p*-values were computed.

#### 2.3.7 Comparison of Models

Internal comparisons of models built under each paradigm were performed, followed by head-to-head comparison(s) of the “winners”. A comprehensive criterion involving both model performance and predictor parsimony was established for the two-stage comparisons. Predictor parsimony stood for both the inclusion of fewer predictors, simpler in functional form, or both. Intuitively, one may deem that including as many predictors as possible in the model and building a model as complex and as close to the true, albeit unknown model of the population by incorporating fancy interaction or nonlinear (e.g., square or cubic) terms in the model will result in better fitting and may hence work better in future populations totally unrelated to the model building process. However, studies have shown that these approaches may lead to overfitting, which means that the model is specially tailored for those in the training set but performs badly for those who are not in the training set, including the vast number of patients in the future clinical setting to whom this model is expected to be of use [20]. On the contrary, incorporating suitably fewer and simpler (e.g., linear and no interaction) terms in the model will, in many cases, lead to better generalizability in future populations, even if the *p*-values of certain predictors are larger than the significance level, which is usually set at 0.05 [20]. However, incorporating too little information in the model will again lead to diminished generalizability in future clinical settings, so the number and functional form of predictors has to be “titrated” to strike a balance. Accordingly, with more emphasis laid on model performance, we chose the model with the highest *c *statistic as the “winner” in both stages of comparison. When there was a tie, the model with more predictor parsimony and hence required less data collection upon clinical application was selected as the “winner”.

## 3. Results

Data from 694 hospitalizations and outpatient visits were used for analyses, with 536 records in the training set and an independent cohort in the validation set. Shown in Table 1, results of univariable analyses are largely consistent with previous research results, with increased age, reduced renal function, increased homocysteine and glycated hemoglobin as well as a larger proportion of male, current smoking, current alcohol drinking, hypertensive and diabetic patients present in patients with CACS >0. Our results also revealed a positive association between current statin use and CACS >0 in both univariable (Table 1) and multivariable analyses (Table 2), which is in line with some (but not all) prior research results that investigated the association of statin use and CAC. A more detailed literature review and discussion concerning this issue can be found in **Supplementary Material Ⅰ**. The number of observations with missing values for each of the predictors demonstrated in Table 1 are displayed in **Supplementary Material Ⅰ**.

**Table 1.** **Clinical characteristics and univariable analyses**.

Predictors*		CACS = 0 (*n *= 262)	CACS >0 (*n *= 274)	*p* value for intergroup differences*	*p* value for nonlinear associations*
Age, years		50.89 ± 9.18	58.70 ± 10.91	<0.0001	0.9994
Gender	Female	85 (32.4)	56 (20.4)	0.0016	-
	Male	177 (67.6)	218 (79.6)		
Hypertension	No	126 (48.1)	89 (32.5)	0.0002	-
	Yes	136 (51.9)	185 (67.5)		
Diabetes	No	216 (82.4)	188 (68.9)	0.0003	-
	Yes	46 (17.6)	85 (31.1)		
Current smoking	No	170 (70.5)	167 (64.0)	0.1183	-
	Yes	71 (29.5)	94 (36.0)		
Current alcohol drinking	Non or occasional alcohol drinker	193 (76.9)	181 (69.6)	0.0634	-
	Regular alcohol drinker	58 (23.1)	79 (30.4)		
Aspirin^†^	No	212 (83.5)	173 (64.1)	<0.0001	-
	Yes	42 (16.5)	97 (35.9)		
Statins^†^	No	208 (81.9)	179 (66.3)	<0.0001	-
	Yes	46 (18.1)	91 (33.7)		
Alkaline phosphatase, IU/L		63.51 ± 18.03	65.97 ± 19.06	0.1370	0.9947
Serum calcium, mmol/L		2.32 ± 0.16	2.33 ± 0.11	0.2753	0.6703
Serum phosphorus, mmol/L		1.17 ± 0.21	1.15 ± 0.16	0.2938	0.4118
Product of serum calcium and serum phosphorus, (mmol/L)^2^		2.69 ± 0.42	2.68 ± 0.40	0.8106	0.9997
Serum creatinine, μmol/L		77.37 ± 16.30	80.61 ± 15.75	0.0196	0.0587
eGFR, mL/(min·1.73 m^2^)		92.91 ± 14.35	86.93 ± 15.44	<0.0001	0.9996
Total cholesterol, mmol/L		4.75 ± 1.19	4.60 ± 1.16	0.1323	0.5249
Homocysteine, μmol/L		12.86 (10.67, 15.49)	14.00 (11.42, 17.79)	0.0003	0.5837
Glycated hemoglobin A1c, %		5.70 (5.40, 6.00)	5.90 (5.60, 6.60)	<0.0001	0.4044
NT-proBNP, pg/mL		35.60 (15.50, 76.30)	60.50 (27.50, 165.70)	<0.0001	0.0261
Lipoprotein(a), mg/L		99.78 (51.03, 235.33)	126.82 (50.38, 303.86)	0.2416	0.5469
Triglyceride, mmol/L		1.59 (1.14, 2.41)	1.48 (1.03, 2.32)	0.1761	0.9959
HDL-C, mmol/L		1.17 (1.00, 1.41)	1.12 (0.96, 1.36)	0.0421	0.9995
LDL-C, mmol/L		2.80 (2.19, 3.39)	2.72 (2.11, 3.36)	0.5364	0.3140
LDL-C/HDL-C		2.39 (1.72, 2.97)	2.43 (1.78, 3.07)	0.4059	0.9990

CACS, coronary artery calcification score; eGFR, estimated glomerular filtration rate; NT-proBNP, N-terminal pro-B-type natriuretic peptide; HDL-C, high density lipoprotein cholesterol; LDL-C, low density lipoprotein cholesterol. *For continuous predictors, values are shown as Mean ± SD or Median (25th percentile, 75th percentile), intergroup *p *values are results of *t *tests or Wilcoxon sum of rank tests, depending on normality. For categorical predictors, data are shown in *n* (percentage), intergroup *p *values are results of χ^2^ tests. Hypothesis testing results of nonlinear associations using generalized additive models are shown in the last column. ^†^“Aspirin” and “Statins” stand for usage of the two medications in the past 7 days prior to laboratory tests.

**Table 2.** **Model results: predictors, coefficients and optimal cut-offs**.

Modeling paradigm	Model No.	Predictors	Predictor coefficients	Standard errors	*p* values*	Optimal cut-off^†^
Paradigm 1	Model 1	Intercept	–1.7636	0.2835	<0.0001	0.50
		Current smoking	0.2062	0.2225	0.3540	
		LDL-C ≥2.95 mmol/L	0.3757	0.2152	0.0808	
		Statins	0.5903	0.3128	0.0591	
		Male gender	1.1187	0.2650	<0.0001	
		Age ≥65 years	1.8871	0.3039	<0.0001	
		Diabetes	0.5637	0.2414	0.0195	
		Aspirin	0.6997	0.3029	0.0209	
	Model 2	Intercept	–1.9268	0.2990	<0.0001	0.42 or 0.43
		Current smoking	0.6125	0.2974	0.0395	
		LDL-C ≥2.95 mmol/L	0.6882	0.2637	0.0091	
		LDL-C*Current smoking	–0.8915	0.4268	0.0367	
		Statins^‡^	0.6440	0.3173	0.0424	
		Male gender	1.1366	0.2657	<0.0001	
		Age ≥65 years	1.9376	0.3074	<0.0001	
		Diabetes	0.5638	0.2431	0.0204	
		Aspirin^‡^	0.6523	0.3065	0.0333	
	Model 3	Intercept	–2.2489	0.3354	<0.0001	0.48
		Current smoking	0.6377	0.2994	0.0331	
		LDL-C ≥2.95 mmol/L	0.6343	0.2661	0.0171	
		LDL-C*Current smoking	–0.8658	0.4301	0.0441	
		Statins^‡^	0.5925	0.3185	0.0628	
		Male gender	1.1071	0.2672	<0.0001	
		Age ≥65 years	1.7560	0.3163	<0.0001	
		Diabetes	0.5828	0.2451	0.0174	
		Aspirin^‡^	0.6585	0.3077	0.0323	
		eGFR ≤100 mL/(min·1.73 m^2^)	0.5435	0.2331	0.0197	
Paradigm 2	Model 4^§^	Intercept	–2.8466 (–4.3860)	0.3597	<0.0001	0.44
		Male gender	1.3231	0.2715	<0.0001	
		Current smoking	0.3915	0.2326	0.0923	
		LDL-C/HDL-C	0.0461 (0.0471)	0.0265	0.0819	
		Age/10	0.2806 (0.2593)	0.0370	<0.0001	
		Diabetes	0.5519	0.2393	0.0211	
		eGFR ≤100 mL/(min·1.73 m^2^)	0.0777	0.2521	0.7579	
	Model 5^§^	Intercept	–2.7576 (–4.1614)	0.3597	<0.0001	0.49
		Male gender	1.3643	0.2702	<0.0001	
		Current smoking	0.4186	0.2317	0.0709	
		LDL-C	0.0105 (0.0112)	0.0254	0.6789	
		Age/10	0.2706 (0.2501)	0.0363	<0.0001	
		Diabetes	0.5502	0.2387	0.0211	
		eGFR ≤100 mL/(min·1.73 m^2^)	0.0947	0.2515	0.7065	

eGFR, estimated glomerular filtration rate; HDL-C, high-density lipoprotein cholesterol; LDL-C, low-density lipoprotein cholesterol; No, number. *p values are results of Wald χ^2^ tests. ^†^Cut-off posterior probabilities are searched among a grid of posterior probabilities with an interval of 0.01, with the one with the highest Youden index selected as the optimal cut-off. Posterior probabilities sharing the same highest Youden index are simultaneously reported. ^‡^“Aspirin” and “Statins” stand for usage of the two medications in the past 7 days prior to laboratory tests. ^§^Some predictors in the models were rescaled in the modeling process to tackle collinearity. For ease of use, coefficients of predictors and the intercepts with the predictors transformed back into their original scale are reported in parentheses.

Collinearity and associated ill-conditioning of the Fisher information matrix were examined for each model built. No collinearity and its associated ill-conditioning were found in Models 1, 2 and 3 whereas both of them were found in Models 4 and 5.

Results of the five models built are detailed in Table 2. When collinearity and ill-conditioning of information matrices were detected, predictors were rescaled to address the problem, resulting in the calculation of coefficients of rescaled predictors rather than those of the original scale. While predictor coefficients of the rescaled predictors are retained in Table 2, coefficients of predictors in their original scale are also reported for ease of use in later clinical settings. A more parsimonious and simpler model (Model 1) was built first, followed by the inclusion of the interaction between current smoking and LDL-C, resulting in a model with slightly better apparent discriminative ability (Model 2 apparent *c *statistic 0.74 vs. Model 1 apparent *c *statistic 0.73). Further inclusion of renal function (eGFR) in the model with dichotomization at 100 mL/(min·1.73 m^2^) increased the model’s apparent discriminatory ability (Model 3 apparent *c *statistic 0.75). In Paradigm 2, medication histories were discarded and dichotomization was averted. Results of the resultant Model 4 and 5 found both models demonstrating better apparent discriminatory ability than the ones built under Paradigm 1 (Model 4 and 5 apparent *c *statistic 0.76) despite the presence of insignificance of several predictors at the 0.05 level. Youden indices of two posterior probabilities were equal in Model 2 and are hence both reported so users can choose either of them as a cut-off.

Numerical validation and calibration results are shown in Table 3. Optimism of performance was calculated via 2000 bootstrap samples, which are subtracted from apparent performance to generate their optimism-corrected counterparts. At internal validation, ranks of the three models built with Paradigm 1 changed. Model 3 exhibited the largest optimism in performance, yet its optimism-adjusted performance ranked the second because of its largest apparent *c *statistic. The relative ranks of Model 1 and 2 remain unchanged due to their similar optimism in *c *statistic. However, none of the three models demonstrated a better discriminatory ability compared to the last two models upon internal validation, with optimism-adjusted *c *statistics of both models equaling 0.75. During independent temporal validation, *c *statistic of Model 1 topped among the first three models, but was still somewhat lower than that of Models 4 and 5.

**Table 3.** **Model results: internal and independent temporal validation, calibration**.

Modeling paradigm	Model No.	Predictors	*C *statistic			Calibration					
			Apparent	Optimism adjusted	Independent temporal validation	Calibration in-the-large		Calibration coefficients		*p* values^*^ of calibration tests	
						Calibration intercept (95% CI)^†^	*p *value^*^	Calibration intercept (95% CI)^†^	Calibration slope (95% CI)^†^	Weak calibration	Calibration slope
Paradigm 1	Model 1	Current smoking LDL-C (dichotomized) Statins Gender Age (dichotomized) Diabetes Aspirin	0.73	0.72	0.77	0.19 (–0.19, 0.57)	0.33	0.22 (–0.18, 0.63)	1.16 (0.70, 1.62)	0.50	0.49
	Model 2	Current smoking LDL-C (dichotomized) Statins Gender Age (dichotomized) Diabetes Aspirin LDL-C*Current smoking	0.74	0.73	0.76	0.18 (–0.20, 0.56)	0.35	0.20 (–0.20, 0.60)	1.08 (0.64, 1.52)	0.62	0.71
	Model 3	Current smoking LDL-C (dichotomized) Statins Gender Age (dichotomized) Diabetes Aspirin eGFR (dichotomized)	0.75	0.73	0.75	0.34 (–0.05, 0.73)	0.08	0.32 (–0.09, 0.73)	0.95 (0.55, 1.35)	0.22	0.81
Paradigm 2	Model 4	Gender Current smoking LDL-C/HDL-C Age/10 Diabetes eGFR (dichotomized)	0.76	0.75	0.79	0.33 (–0.07, 0.74)	0.10	0.32 (–0.08, 0.71)	0.87 (0.54, 1.20)	0.18	0.43
Paradigm 2	Model 5	Gender Current smoking LDL-C Age/10 Diabetes eGFR (dichotomized)	0.76	0.75	0.79	0.31 (–0.09, 0.71)	0.13	0.29 (–0.10, 0.68)	0.86 (0.53, 1.19)	0.20	0.40

eGFR, estimated glomerular filtration rate; HDL-C, high density lipoprotein cholesterol; LDL-C, low density lipoprotein cholesterol; No., number; CI, confidence interval. *p values are results of Wald χ^2^ tests. †Wald confidence intervals are calculated and reported.

**Fig. 1.**
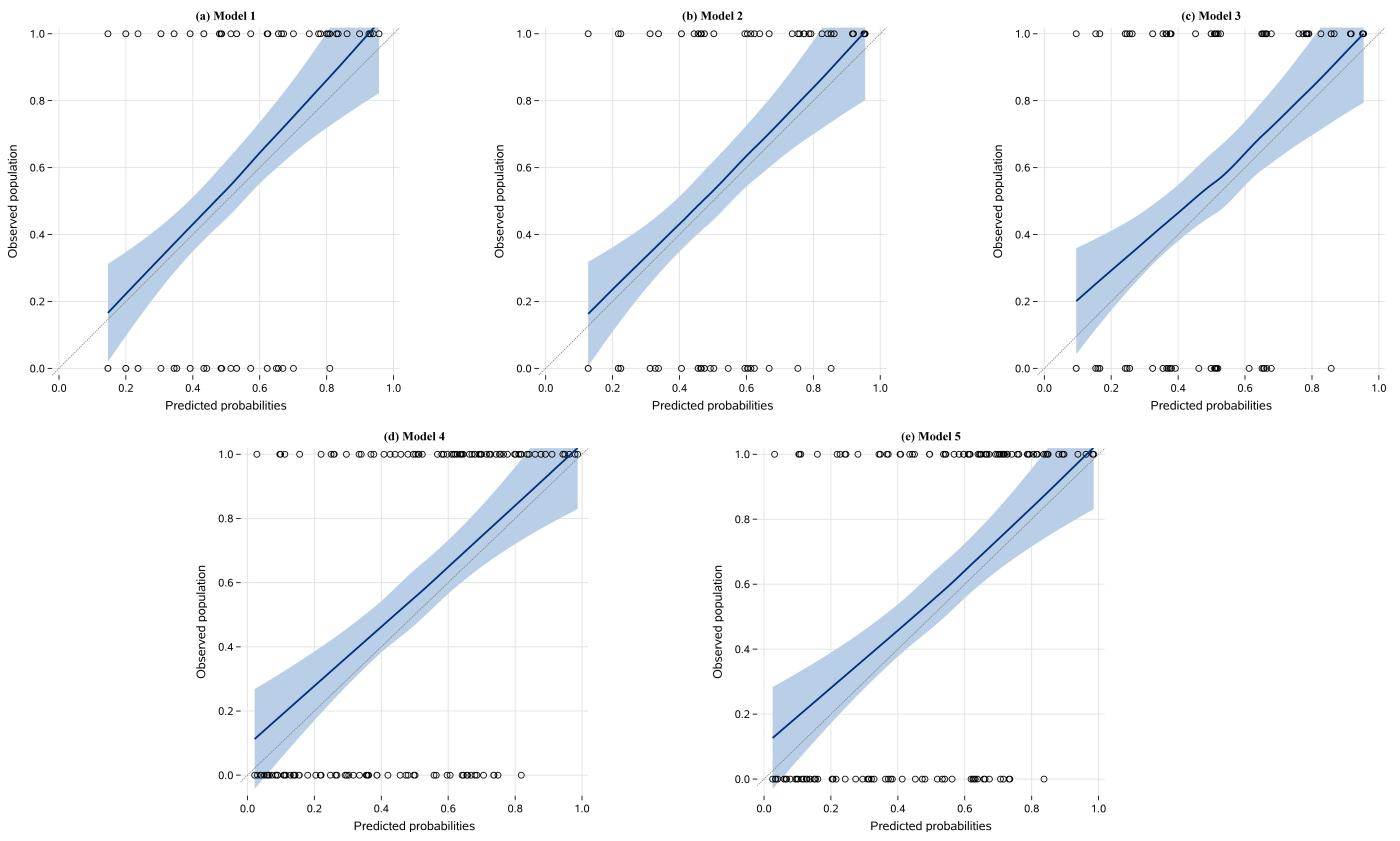
**Calibration plots of models**. (a): calibration plot of Model 1; (b): calibration plot of Model 2; (c): calibration plot of Model 3; (d): calibration plot of Model 4; (e): calibration plot of Model 5. The x-axis of each panel represents patients’ predicted probabilities calculated with the corresponding model while the Y-axis shows actual presence or absence of CAC in patients. Black circles are data points of scatterplots of the duo. Dashed diagonal lines are reference lines of “perfect calibration” ( y=x ) while dark blue solid lines are locally weighted scatterplot smoothing (*loess*)-smoothed calibration curves, which are enclosed by light blue confidence bands. CAC, coronary artery calcification.

Using locally weighted scatterplot smoothing (*loess*), a popular scatterplot smoothing method as smoothers, calibration plot of each model was drawn and displayed in Fig. 1. Results indicated good calibration of all models.

To further facilitate application of the models by users, a spreadsheet was developed for the calculation of probability for having CAC and is provided as **Supplementary Material Ⅱ**. The spreadsheet is capable of calculating the probability of having CAC via Model 5, as it was the best performing model developed by the DEPICT study and is the one recommended for further validation and application. Brief yet sufficient introduction has been added to the spreadsheet, allowing automatic assessment of whether the subject is afflicted by CAC after typing in his or her information.

## 4. Discussions

To enable radiation-free evaluation of CAC, the DEPICT study, was carried out with gender, current smoking, LDL-C, age, diabetes and reduced renal function as potential predictors. Under two modeling paradigms-one with a larger number of predictors, more processing of their original information and a more complex layout of the configuration of the models and one with slightly fewer predictors, more preservation of the original information of predictors, as well as less elaborate model configurations- five models were built. Internal and external validation confirmed Model 5—a model built under Paradigm 2—outperformed all other models in terms of discriminative ability. All models built are well-calibrated. Therefore, Model 5 is recommended for further application in clinical practice. A patient’s predicted (i.e., posterior) probability of having CAC can be obtained by performing calculations using the spreadsheet provided. A predicted probability of at least 0.49 is indicative of having CAC.

As highlighted in the introduction section, the objective of this study was to build clinical prediction models capable of telling the doctor whether an individual has CAC (or not) using medical history and common, non-invasive test results with an acceptable assessment accuracy. Despite the English word “predict” carrying the subtle meaning of determining *the future *rather than *the present*, the idea of prediction be generalized to settings where doctors and patients wish to know if a disease were present without needing to undergo sophisticated exams, even at the cost of an acceptable loss of precision. Calculation of eGFR and fingertip blood glucose testing are all exemplifications of this scenario. In the very specific case of CAC, despite the presence of many imaging modalities capable of detecting CAC, many of them (e.g., coronary angiography) may be fraught with bias associated with decreased sensitivity, specificity as well as the confounding effect of interobserver variability. Furthermore, CT is the only non-invasive modality capable of quantifying CAC, yet repetitively performing CT with the sole purpose of acquiring CACS and using these results to unravel how CACS can further be incorporated into clinical practice (e.g., examining whether a drastic increase of CACS in three months warrants immediate clinical intervention) is hardly justifiable as competing modalities like coronary angiography carry the benefit of being able to tackle coronary lesions while assessing CAC. Development of new modalities with low or no radiation and can hence be repetitively performed is a solution. However, our study took a different approach- rather than attempting to develop a new modality, we built models that lower the radiation dose required to assess CAC to zero.

Prior research has revealed the heterogeneities between those with and without CAC [8,9,10,11,12,13,14,15], justifying the practice of building models that effectively distinguish them. In fact, patients with CACS = 0 accounted for almost half (48.88%) of the patients in the entire training set. This is in line with previous research findings [27], indicating that the plurality of patients with CACS = 0 in our dataset is not the result of a biased sampling process. From a statistical perspective, CACS data is zero-inflated (or semi-continuous). While already devoting much of the contents of this research paper to intricacies in various statistical and machine learning topics, we wish to brief convey the message that zero-inflated data should be modeled with tailored statistical methods by now. Trying to break the entirety of the data into the zero and non-zero portions and build separate models where information on one portion is almost completely ignored in the modeling process of the other will result in models with erroneously calculated regression coefficients and therefore produce biased prediction results upon application [28]. Despite the presence of statistical methods of modeling zero-inflated data, much of the attention of statisticians in this field has been on determining the association between independent variable(s) and the dependent variable (especially on the special occasion where the dependent variable is discrete instead of continuous in nature) while methodological research on discriminative (e.g., the like of *c *statistic) and calibration methodologies—two fundamental aspects of building models for prediction instead of merely unraveling associations—are limited. It is unclear whether measures of discriminative performance in binary classification settings can be directly applied to zero-inflated models with sound theoretical support for their plausibility. The more intractable problems arise in calibrating zero-inflated models. Despite the authors’ extensive search for statistical literature, guidelines on the way to calibrate zero-inflated models were not retrieved. This comes as no surprise to the authors after contemplation of the problem’s complexity. Given the zero-inflatednature of the dependent variable, it is natural that both the observed and predicted CACS have a large portion of zeros and that misclassification such as falsely classifying a patient without CAC as a subject that has it and therefore calculating a positive CACS for him/her, and *vice versa*, can take place. Now let us try to calibrate the model by plotting the predicted CACS against the observed counterpart like we do in calibrating multiple regression models. It is disturbing to note that due to the almost inevitable misclassification, some subjects will have a “real” CACS of zero and a predicted CACS larger than zero, while some will have a “real” CACS larger than zero and a predicted one precisely equal to zero. Therefore, the scatterplot of "real" vs. predicted CACS will have dots aligned along both the horizontal and the vertical axis. What is more, there is no guarantee that misclassification happens to all subjects with a particular range of “real” CACS values. Consequently, a patient with a “real” CACS of, say, 20, might be falsely classified as being non-coronary-calcified and therefore have a predicted CACS of zero while another person with a somewhat different risk profile yet identical “real” CACS value (i.e., also 20) will be correctly classified as being CAC-afflicted and gets a predicted CACS of, say, 25. It is also likely that a person with a predicted CACS of 30 has a “real” CACS of zero while another one with the same predicted CACS has a “real” CACS of 35. These complexities culminate in the existence of dots on the scatterplot aligned along lines perfectly vertical to both the horizontal and vertical axes. It is not difficult to envision that there does not exist a straight line that passes through the dots on the scatterplot with reasonable error as it does in scatterplots where the data points are aligned with a straight line. If we force the statistical software to build such lines, the null hypothesis that the line’s slope is equal to one and that its intercept is equal to zero- both signs of perfect calibration- will almost surely be rejected. As a consequence, if we simply copy the practice of calibrating linear regression models when we attempt to calibrate zero-inflated models, we are unlikely to build a well-calibrated model. In addition, it is uncertain whether goodness-of-fit statistics widely used in multiple regression such as the mean squared error can be applied to such models as well. In all, so many methodological uncertainties were found when we attempted to build a model that could not only tell whether a patient has CACS, but could also assess the patient’s CAC severity by calculating a predicted CACS if the model predicts that he/she has CAC. On the contrary, if we restrict our goal to only the first one (i.e., build a model that can only conduct radiation-free diagnosis of CAC but cannot tell how severe it is once it predicts its presence), logistic regression suffices. With regard to this goal, the presence of extensive studies devoted to the details of statistical methodologies regarding using logistic regression for prediction made achieving this goal possible. We therefore confined our goal to distinguishing those with CACS = 0 and CACS >0, which is still of clinical significance.

For instance, if uncertain about initiating statin therapy for asymptomatic individuals aged 40 to 75 years without diabetes, has a low-density lipoprotein cholesterol level of 70 to 189 mg/dL or greater, as well as a borderline (10-year atherosclerotic cardiovascular disease (ASCVD) risk 5.0% to <7.5%) or intermediate risk (10-year ASCVD risk 7.5% to 20%), the American College of Cardiology (ACC)/American Heart Association (AHA) guidelines [29] indicate that suffices it to ascertain whether they have CAC (instead of the exact value of CACS) for further stratification of cardiovascular risk as well as decision on whether statins are recommended [18]. Therefore, our model may serve to facilitate a radiation-free cardiovascular risk re-stratification and decision making in these populations.

The application of the models developed in the DEPICT study may even go beyond the clinical care of CVDs. For instance, it has been found that compared with those with CAC, people free from the disease have a reduced likelihood of hip fracture [7]. Yet, until now, the authors have failed to any retrieve studies that further explore the clinical utility of CAC in the prevention and treatment of hip fracture, including if and when CAC testing should be conducted in individuals at high risk of the disease. As previously stated, conducting chest or coronary CT exams for the prevention of hip fractures presents ethical issues due to the harm caused by radiation exposure. This causes a paucity in samples for researchers attempting to conduct studies on exploring the clinical utility of CAC in hip fractures, which in turn causes a lack of evidence in favor of conducting CAC testing in subjects at high risk of hip fracture, and further exacerbates the ethical concerns on exploring the clinical utility of CAC in hip fractures. In all, a vicious cycle is formed. As long as conducting CAC testing is radiation-dependent and costly, it is hard to terminate the cycle, as two of the most important players in this cycle are not removed. However, the DEPICT study allows patients to ascertain their likelihood of having CAC with no radiation and less costs. Therefore, future researchers can first explore the roles DEPICT model-predicted CAC play in the prevention and treatment of hip fractures. In this way, the clinical utilities of CAC in hip fracture could be unraveled. The same logic applies to other non-CVDs as well, such as cancer, chronic obstructive pulmonary disease and chronic kidney disease, which have all been shown to be related to CAC [7].

However, the DEPICT study is only dedicated to developing and validating models to determine whether a patient is likely to have CAC or not, instead of how severe it is once the patient is predicted by the model to have CAC. Therefore, decisions involving knowing the value of CACS are still not radiation-free. For instance, the ACC/AHA Primary Prevention Guidelines [30] suggest that individuals with CACS ≥100 or a CACS score in the 75th percentile or above be administered statins [18]. Models developed by the DEPICT study are not able to facilitate decision-making in such settings.

The statistics of prediction and examining associations are vastly different. The primary goal of prediction is to build models that work on future populations. Instead, the focus of assessing associations is on explaining the data at hand while less attention is paid to how effectively the conclusions reached can be generalized to other populations. More specifically, despite being counterintuitive to professionals not involved in prediction modeling, *p*-values of the individual terms and sophisticated terms like interaction and nonlinear terms are not as important as they are in building statistical models that only attempt to examine associations. Pursuit of small *p*-values by *p*-value-based variable selection or incorporating interaction and nonlinear terms may result in overfitting and decrease the generalizability of the model in future populations, especially in small samples [20]. Steyerberg [20] elaborated on the potential harm of variable selection in predictive modeling by introducing the concept of testimation bias. Briefly, data at the hand of every researcher are essentially samples of the population on which they wish to analyze. Every modeling process is a de facto estimation of the true, albeit unknown, coefficient of the population based upon the sample data at hand. Disparities exist if the researcher sampled repetitively from the underlying population, with some of the estimated regression coefficients closer to the true value and others further from it. *p*-value-based selection amounts to discarding those predictors with less extreme but closer-to-real regression coefficients and preserving the extreme ones, which deviate more from the true value. Therefore, *p*-value-based variable selection is prone to overfitting, which means that the model is specifically tailored to the current data and does not work well in future individuals from the very population the researcher intended to predict in the first place. More details regarding testimation bias can be found in Steyerberg’s book [20], which also gives practical examples on how incorporating interaction and nonlinear terms in the data might lead to overfitting as well.

To tackle testimation bias and overfitting, Steyerberg [20] suggested that the models be built parsimoniously in predictors, especially when the sample size is not large. The DEPICT study adhered to this suggestion. Despite this, we included a certain degree of complexity and flexibility in our model in an attempt to reflect the real underlying effect of predictors. Examination of nonlinear associations of predictors with logit*p* was conducted. None of the nonlinear associations between each of the candidate predictors and logit*p *were significant. Nonlinear terms were accordingly not included in all models.

In line with Steyerberg’s suggestion [20], candidate model predictors were specified with a rationale largely based on literature review and, to a lesser extent, data-driven results. Steyerberg [20] also suggested avoiding categorization of continuous predictors into discrete ones, a common practice in logistic regression modeling, despite the fact that certain models Steyerberg’s research team and other researchers built and cited in Steyerberg’s book followed the usual categorization approach. The DEPICT study therefore adopted two Paradigms in the modeling process: Paradigm 1, which followed the usual practice of categorizing continuous predictors and included medication history; and Paradigm 2, which discarded all medication history terms to offer alternatives free of potential recall bias and averted dichotomization of predictors under the premise of biological plausibility of the coefficients.

In Paradigm 1, dichotomized age, LDL-C and eGFR, gender, diagnosis of diabetes, as well as use of statins and aspirin were chosen as candidate predictors. A plethora of cardiovascular risk factors are correlated with the use of aspirin and CAC. However, putting all risk factors in the models might lead to overfitting. Use of aspirin therefore acted as a surrogate in the model. Statin use was included because of its potential association with CAC. We would like to reiterate that use of medications was defined as use at least once in the past 7 days prior to laboratory tests, which was a pragmatic consideration given the retrospective nature of our study and real-world clinical scenarios. Medical records of our data often only recorded the prescriptionsand generally lack information on patients’ compliance, not to mention that recall bias may occur when the latter was self-reported. It is also possible in the outpatient setting that patients receive a CT exam first and had laboratory tests taken weeks afterwards. Given the relatively slow progression of CAC (Anand et al. [31] found the mean CACS progression per year to be 16.1 in a population with type 2 diabetes), the relatively rapid impact of statins on lipid levels, and the short time lag (≤one month) defined by the study’s inclusion criteria, we chose pre-laboratory test use of statins to define medication history. Usage of aspirin followed the same definition to lessen doctors’ need to acquire a second version of medication history, avoid confusion in the clinical setting and reduce recall bias. More detailed considerations regarding the specification of predictors are documented in **Supplementary Material Ⅰ**.

Among the models built under Paradigm 1, Model 1 had less flexibility and more parsimony. Models 2 and 3 included interaction in terms of current smoking and elevation of LDL-C to increase flexibility of the model and explore the reason for statistical insignificance of current smoking, resulting in the significance of all predictors at the 0.05 level. This inclusion was largely data driven. At present, its possible biological plausibility may be associated with the intercorrelation of smoking and LDL-C levels. A study by Hallit et al. [32] on 308 Lebanese individuals found a positive association between cigarette smoking and elevated LDL-C. Coexistence of waterpipe and cigarette smoking exacerbated the correlation [32]. Later research results found association between both current and former waterpipe smoking with elevation of LDL-C [33]. A study on 360 Sri Lankan current male tobacco smokers and their 180 male, non-smoking compatriots found a positive association between current smoking and elevated LDL-C [34]. A study on 1504 American adults (58% women, 84% white) found a weak, positive, yet statistically significant association between multiple smoking intensity parameters and elevation of LDL-C [35]. However, a study on 9846 Chinese adults (6774 non-smokers and 3072 smokers) found a significant negative association between smoking and elevated LDL-C [36]. Another study on 707 male adults from northwestern rural China found a positive yet statistically insignificant association between smoking and elevated LDL-C [37]. In all, the majority of studies investigating the relationship between smoking and LDL-C found the intercorrelation between them present, regardless of its quality (positive or negative association). Given the fact that smoking is associated with other pathophysiological processes involved in atherosclerosis (e.g., endothelial dysfunction, disruption of coagulation systems) aside from increased LDL-C, it is plausible that smoking alters the association of LDL-C and CAC.

Model validation ensued model building to test their discriminative performance in future settings. Temporal validation was chosen for external validation. In this process, the ranks of performance statistics of models built under Paradigm 1 changed. In previous stages of the modeling and validation process, an increase in model complexity enhanced apparent performance. In internal validation, more complex models still exhibited better performance among those with similar predictors, with an optimism-adjusted *c *statistic of Model 2 and 3 ranking higher than that of Model 1 (0.73 vs. 0.72). However, the ranking list was reversed in external validation, where Model 1 topped the other two models built under Paradigm 1 in *c *statistic (0.76). This is consistent with Steyerberg’s findings [20] that blindly adding model complexity without regarding sample size is prone to overfitting.

We next adopted Paradigm 2 and built Models 4 and 5. In Model 4, substitution of LDL-C with LDL-C/HDL-C is an attempt to include more effects in the model while scaling age by 10 was an attempt to increase the interpretability of its coefficient. Rescaling of predictors was done prior to the coefficient calculation process due to collinearities of predictors with the intercept but no collinearity among the predictors. Despite the loss of information on medication and statistical insignificance of several predictors at the 0.05 level, aversion of dichotomization resulted in a better discriminatory model (Model 4 apparent *c *statistic 0.76).

In Model 5, we substituted LDL-C/HDL-C with LDL-C to limit the need for the acquisition of data in clinical use. It is somewhat surprising that despite the greatly enlarged *p*-value of lipids, the resulting model’s apparent discriminatory ability remained unaffected (Model 5 apparent *c *statistic 0.76). It is noteworthy that the *c *statistics (rounded to 2 decimal places) were identical in both internal and external validation, signifying the slim impact of inflation of *p*-values in certain predictors on model performances as well as the limited contribution of HDL-C to model discriminative ability.

Calibration of models revealed that all the models built were well calibrated, with no systematic over- or underestimation of probabilities and no overfitting. Graphical results revealed that the shapes of all calibration curves were straight lines closely adjacent to and sharing a similar slope with the diagonal line, which was enclosed in confidence bands of all models. These results are consistent with various calibration hypotheses testing results and CIs, with all of the tests in Table 3 statistically insignificant at the 0.05 level, all the CIs of calibration intercepts enclosing 0 and those of calibration slopes enclosing 1.

Head-to-head comparison of the “best” models built under the two paradigms was performed. Both Model 4 and 5 outperformed the “best” model built under Paradigm 1 in terms of apparent, optimism-adjusted and temporally validated *c *statistic. Since all of the models were well calibrated, the comparison of the “best” models in terms of calibration provided limited information. Given the parsimony of Model 5, it was recommended for further validation and use.

Currently, few researchers have focused on the limitations of CT in the acquisition of CACS and have attempted to resolve them. Among those research projects that have something to do with this problem, few of them underwent standard procedures necessary for proving that the model built is potentially applicable in future clinical settings (i.e., stringent internal and external validation).

Fan et al. [38] built two models capable of “predicting” CAC-one without restriction and the other with the aim of “predicting” dual presence of coronary artery disease (CAD) and CAC with data of 562 patients. However, the article did not describe how the entire dataset was partitioned into the training and validation sets and if internal and external validation were conducted. Therefore, it was assumed that the entire modeling and validation process was conducted on the entire sample. This, as previously described, will cause the performance of the prediction model to be influenced (usually inflated) by optimism [20]. In other words, it is unclear whether they can perform as well as is demonstrated in the paper. Moreover, even the optimism-inflated discriminative ability of the models (*c *statistics: 0.728 for the model predicting presence of CAC, 0.717 for the model predicting dual presence of CAC and coronary artery disease) are lower than that of Model 5 in the DEPICT study. In addition, despite the proclaimed goodness-of-calibration of the models, they were all calibrated on data from the training set. Steyerberg [20] voiced explicit objection against this procedure, as it, along with calibrating the model during internal validation, is uninformative in that the results will always be good. Also, no hypothesis testing was conducted for calibration, meaning no numerical results were presented to support the claim that the models were well-calibrated. In all, compared with the study by Fan et al. [38], the DEPICT study produced models with better performance and more promising applicative potential for users.

Park et al. [39] also built models predicting presence of CAC, but in a Korean population. The size of the entire sample (3302) was larger than that of the DEPICT study, yet due to the design of the study, the number of samples used for training was small. Park et al. [39] adopted a rather peculiar way of building and validating models- they first split the dataset into the training and test set, and then further partitioned the training set into ten folds. In each fold, 90% of the data were used for training while the remaining 10% was used for validation. The modeling process was conducted on each fold’s training section and validated in the validation part of the same fold. In other words, only around 238 records were used for the modeling process of every model generated in the study by Park et al. [39]. This greatly increases the instability (in statistical terms, variance) of the estimated parameters of the model, considering that not only logistic regression, but also several data-hungry modeling methods like CART and several of its variants were used. Moreover, the role the testing set plays in the development and validation of the models was not mentioned in the entire manuscript and it is not clear if the final results presented were based on the validation part of the same fold or on the test set. Putting these issues aside, the models built by Park et al. [39] demonstrated good performance, with the best model having a *c *statistic of 0.765, slightly lower than that of Model 5 of the DEPICT study under external validation. The logistic regression model built by Park et al. [39] appeared well-calibrated as well. Another fundamental issue for the study by Park et al. [39] is the lack of methodological theoretical support for the design. As has been said, the study by Park et al. [39] adopted a rather peculiar design that essentially skipped internal validation. While wishing to stress that we do not think it is a ‘must’ but is rather a ‘should’, we would also like to announce our unawareness of any statistical literature endorsing the design by Park et al. [39], which not only encompasses skipping internal validation, but also breaking the training set into folds and further breaking the folds into training and validation portions, as well as building exactly 100 models and selecting the best one, without mentioning the multiplicity problem of hypothesis testing. The entire Park et al. [39] paper made no mention of the statistical plausibility of such procedures either. However, the study by Park et al. [39] was conducted on data from two centers, which is an advantage over the DEPICT study. To summarize, despite methodological concerns, the study by Park et al. [39] resulted in well-performed models with discriminative ability competitive to the best model of the DEPICT study. In addition, the DEPICT study was conducted on Chinese populations and can be anticipated to perform better than the model built by Park et al. [39] in Chinese populations.

An aside to note before we conclude the discussion on the research project by Park et al. [39] is on the rank of the performance of models built with different techniques. It is interesting to find that despite their attractive names and popularity among non-professionals of statistics and artificial intelligence (AI), tree-based methods like CART, conditional inference tree (CIT) and random forest, all exhibited discriminative performance inferior to that of logistic regression, a method that has been deemed too simple and old-fashioned by some professionals in fields where AI has a role to play but not necessarily AI professionals themselves. In all, the results of the study by Park et al. [39] are vivid exemplifications of our foregoing summarizations of comparative research on the performance of logistic regression and various other modeling techniques with attractive names and good performance in non-medical domains.

Strengths of the DEPICT study include: (1) Using medical history and common, non-invasive test results, we built and validated (both on the training set and on an independent sample) mathematical models capable of assessing the presence of coronary calcification (CAC) without CT scans upon use. (2) Our model enabled cheap, convenient, frequent, radiation-free assessment of the presence of CAC. (3) Our model lowers the cost of repetitive assessment of CAC, provides a platform for exploring clinical utilities of CAC that have remain unexplored, culminating in the integration of CAC into cardiovascular and non-cardiovascular care. (4) We provide an approach to radiation-free assessment of atherosclerosis in the era of big data and an approach to the research paradigm on the integration of data science and clinical medicine for better, personalized health care, thereby providing inspiration for future research directions to health care providers, statisticians and mathematicians.

### Limitations

However, the DEPICT study also has its limitations, including: (1) The DEPICT study was conducted in a single center. (2) The DEPICT study is retrospective in nature. (3) The coexistence of the issues mentioned in (1) and (2) might have contributed to samples with selection bias, leading to unknown generalizability in other clinical settings.

## 5. Conclusions

Under two paradigms, five logistic regression models were built to assess the presence of CAC. All models were well calibrated. When discriminatory performance and parsimony were used as criteria of superiority, Model 5 had the highest *c *statistic and required less information than its competitive counterpart. It was therefore recommended for further validation by other researchers and utilization in real-world settings.

## Data Availability

Data of the current study could be made available in response to a reasonable request proposed to the corresponding author.
