# Supplementary material for: Development of Mathematical Models Evaluating Presence of Coronary Calcification Independent of Computed Tomography (DEPICT): Radiation-Free Evaluation of Coronary Atherosclerosis
Source: Rev Cardiovasc Med. 2026 Jun 15;27(6):46777. doi: 10.31083/RCM46777 (PMC13339184; doi:10.31083/RCM46777)
Supplement: Supplementary file 1 [file 2153-8174-27-6-46777-s1.zip › Second reviseion in May-Supplementary Material I.docx]

**Supplementary Material Ⅰ**

**of**

***Development of mathematical models Evaluating Presence of coronary calcification Independent of Computed Tomography (DEPICT): Radiation-free evaluation of coronary atherosclerosis***

1. **Rationale of selection of predictors**

The following paragraphs are detailed descriptions of considerations regarding specification of predictors. Before we begin our elaboration regarding each variable, we would like to briefly review the complexity of vascular calcification as well as its implication on our study, with the emphasis on coronary artery calcification (CAC).

Generally speaking, vascular calcification itself is a heterogeneous disease, with subtypes present when location is used to as the categorization rule. In this sense, the popular way of categorization of vascular calcification is intimal and medial calcification[1], while some take both location and mechanism of vascular calcification into consideration and hence classify vascular calcification into atherosclerotic intimal calcification, medial calcification and genetic calcification[1-2]. Readers might notice the similarities of the nomenclature of different subtypes of calcification according to the two categorization rules and hence conjecture that intimal calcification is closely related to atherosclerosis and medial calcification is not. That is indeed the case. Atherosclerosis is indeed closely related to intimal calcification, with inflammatory mediators playing important roles in this process. On the other hand, medial calcification may be associated with chronic kidney disease or genetic disorders like Marfan syndrome, with metabolic factors (e.g. phosphate, uremic toxins, glucose) mediating the calcification process in the former circumstance and genetic factors (defects in various genes like *OPG*) mediating the process in the latter circumstance[1]. What complicates the situation in real-world clinical settings is that risk factors for both intimal and medial calcification may coexist and exert influences reciprocally, making it possible for the existence of paradoxes between a priori deduction of the possible subtype of vascular calcification and what we actually observe in real world. Readers will see examples of the paradoxes we mention here in the following paragraphs.

Features of imaging modalities complicate our variable selection process. Despite the coexistence of both intimal and medial calcification in coronary arteries, calcification ascertained by computed tomography (CT) is in general intimal calcification, with research demonstrating the ability of electron beam CT to detect small intimal calcium deposits that correlate with early atherosclerosis when no luminal narrowing was observed[3]. Coronary calcium detected upon CT has therefore been regarded as a marker of coronary atherosclerosis[1]. But by no means that this can be translated as the risk factors for medial calcification are of significance and should therefore not had been included in the models. We will give examples on this in our later discussions.

Despite the inherent heterogeneities between the two subtypes of vascular calcification, clinically popular and routinely performed imaging modalities like CT are not capable of quantify the two kinds of vascular calcifications via means like separating CACS into intimal CACS and medial CACS. Nevertheless, as is cited above, a gross assessment of coronary calcification via CACS is capable of identifying those with higher risks and those who receive more benefit from statin therapy, both in a quantitative (exact number of CACS or the group a patient belongs to following grouping of patients’ CACS by doctors) and qualitative (CACS=0 or CACS>0) manner. All in all, the inherent heterogeneities of different subtypes of vascular calcification have no impact on the clinical utility of CACS and the necessity of correctly distinguishing patients whose CACS=0 and those CACS is larger than 0. What has been of concern in the variable selection process before statistical variable selection is that despite the close associations between atherosclerosis and vascular calcification, results of studies investigating the associations between some acknowledged risk factors of atherosclerosis and vascular calcification are not always consistent. In addition, some studies explicitly pointing out the subtype(s) of vascular calcification they analyzed while others did not. We took both results of literatures and expert opinions into consideration in the course of selection. The candidate predictors as well as the reasons why they were eventually specified in multivariate modelling are as follows.

**Current smoking**: Current smoking is defined as smoking within the last 1 month prior to taking CT exam. Smoking is an acknowledged risk factor for atherosclerosis. When it comes to the association between smoking and arterial calcification, disparities in the research results exist. As is stated above, intimal calcification is closely related to atherosclerosis, which in turn is closely associated with smoking[1]. A study investigating autopsy patients revealed that smoking is a risk factor of coronary calcification[4]. We pause here to remind readers of another discovery of this study, which exemplifies the paradoxes between a priori deduction of the exact subtype of calcific and what we observe in reality. The researchers found that while the study population encompassed patients of a broad range of kidney function, the prevalence and severity of coronary intimal calcification was more pronounced in chronic kidney disease (CKD) 5D group than those in the CKD 4/5 and CKD 3 groups. This discovery is against what we would expect from a priori thinking based on the fact that impaired renal function is associated with medial rather than intimal calcification. However, when dyslipidemia often coincides with impaired renal function is also considered in our thinking, this finding no longer takes us by surprise. In this sense, impaired renal function is not only associated with medial, but also with intimal calcification. Starkman et al. also found that prevalence of CAC in young (not older than 28 years old) smokers with type-1 diabetes mellitus (DM) was greater than that of type-1 DM patients that did not smoke[5]. We would like to pause again to point out the prevalence (10.9%) of coronary calcification (measured as CACS>0) in this cohort that comprised totally of subjects aged between 17 to 29 years old and have at least a history of type-1 DM for 5 years. This finding serves as another example of the paradoxes we mentioned earlier, as diabetes is associated with medial calcification, but as we have mentioned earlier, CACS is primarily a marker of intimal calcification and atherosclerosis. This also reminds us that risk factors of medial rather than intimal calcification should not be excluded from our model. On the other hand, the association between medial calcification and smoking is less robust. Histological studies conducted on 431 superficial femoral artery samples from organ donors revealed negative association between smoking and medial calcification of the samples[6]. As for the studies that did not separately explore the associations between intimal or medial calcification and smoking but rather considered calcification as a whole, a preponderance of studies revealing the positive association between smoking and vascular calcification is observed. A study on 409 individuals revealed the correlation between smoking and ^18^F-sodium fluoride (^18^F-NaF) uptake of the femoral artery during positron emission tomography/CT (PET/CT)[7], a modality capable of identifying microcalcification in the lower limbs[8]. In another study, analysis of 334 saphenous vein graft lesions by intravascular ultrasound (IVUS) identified post-coronary artery bypass smoking as an independent predictor of saphenous vein graft calcification[9]. More recently, using data of 211 subjects that underwent enhanced chest-abdomen-pelvis CT angiography and a modern machine learning method named random forests, Jadidi et al.[10] explored the risk factors of vascular calcification in both a general and vascular zone-specific manner. They found that total calcification was more pronounced in former and current smokers than those who never smoked. Among the vascular zones examined, such an association was only statistically significant in the abdominal aorta. Unfortunately, coronary arteries were not investigated in this study. Eventually, given the acknowledged fact that smoking is a risk factor of atherosclerosis, the close correlation between both intimal calcification and smoking as well as CACS and coronary intimal calcification and atherosclerosis, we included smoking in our models. We next decided to include *current* smoking rather than *current or former* smoking in the models for the following reasons. Heterogeneity of the time from smoking cessation to taking the CT exam lies in the population who former smoked but later quitted. Adding the duration of smoking cessation in the model was a solution, but bias in recalling the duration of smoking cessation posed a problem. Addition of smoking cessation duration in the model also consumes extra degrees of freedom, which may cause overfitting. A study also shows the lack of association between former smokers and CACS>0[11], which may suggest a less important role of former smoking in the prediction of CACS>0. Therefore, we put current smoking in the model in the end.

**LDL-C**: LDL-C has been acknowledged as an important risk factor in atherosclerosis. Endothelial dysfunction enables accumulation of LDL-C particles in the arterial wall, in which LDL-C particles are further oxidized, triggering inflammatory response. Monocytes then enter the arterial wall, in which they convert into oxidized LDL-C and engulf them, forming foam cells that lead to the formation of fibroatheromas[12]. Calcification follows this process. Plaque components including apoptotic cells, extracellular matrix and necrotic core may be calcified[13]. There is also clinical evidence demonstrating the positive association between elevation of LDL-C and CAC. Hao et al.[14] conducted a study analyzing data of 6544 atherosclerotic disease-free individuals (58.2% were white) and found that elevation of LDL-C was an independent risk factor of CAC progression after adjustment of other traditional risk factors. Of note, they defined CAC progression as a blend mixture of events that encompassed transition of CAC=0 at baseline into CAC>0 at follow-up, which is most relevant to our study.

However, there are also other evidences casting doubt on the positive correlation between elevated LDL-C and vascular calcification. A detail interesting to note is that among 4085 people aged between 45 to 84 years old and met enrolment criteria of randomized clinical trials (RCTs) of statin therapy in the Multi-ethnic study of atherosclerosis (MESA) study, nearly half had a CACS of 0[15]. In addition, Hong et al. conducted a study[16] investigating 625 lipid-lowering agent naïve Korean patients with LDL-C not higher than 135 mg/dL (3.50mmol/L) did not find statistical significance among of LDL-C levels among patients whose CACS=0, 0<CACS<400 and CACS not smaller than 400. Similar results were also found when patients were categorized into CACS=0, CACS>0 and CAC< 90^th^ percentile as well as CACS not smaller than 90^th^ percentile[16]. As for the very specific problem we wished to tackle, Hong et al. found that elevation of LDL-C was negatively associated with CACS>0 in univariable analyses, despite the statistical insignificance of this association. Their study also found that association between elevation of LDL-C and neither CACS≥400 nor CAC≥90^th^ percentile were statistically significant. A RCT involving 471 patients found no association between on-statin-treatment LDL-C and CAC progression (defined as percent change of the total CAC volume score) in the pooled population[17]. Given the close association between LDL-C and atherosclerosis, the close association between atherosclerosis and intimal calcification and the fact that CT scans predominantly reflect intimal calcification, LDL-C was included in our models.

**Interaction of smoking and LDL-C**: Admittedly, despite the acknowledged contributions of both smoking and LDL-C to atherosclerosis, few studies have investigated their interaction or their respective effect on atherosclerosis or vascular calcification when stratified by the other predictor. A study, though, did reveal the interaction of the effects of smoking and LDL-C on the incidence of coronary heart disease (CHD)[18]. Given the close correlation between CHD and coronary calcification, and the fact that both terms of current cigarette smoking and higher levels of LDL-C were statistically insignificant in our tentative multivariate logistic regression modelling had this interaction term not been added but were both statistically significant in the presence of this interaction term when *p*<0.1 was used as the level of statistical significance. The interaction term itself was statistically significant too in the multivariable regression model, as demonstrated in Table 4.

**Current use of statins**: This is defined as usage of statins within the past 7 days prior to taking CT exam. While statins have been proved to be beneficial to atherosclerosis, a large body of evidence suggest the positive association between statin and CAC. In 2012, Nakazato et al. found the association between increase in calcific plaques and statin therapy[19]. In 2015, a post-hoc analysis found that high-intensity statin therapy is associated with an increase in CAC, despite a reduction on the progression of plaques[20]. Our study found that statins was statistically significantly associated with CACS>0 in both univariable and multivariable analyses. We therefore included use of statins in our models.

**Gender**: Gender has been for a long time been recognized as a significant factor in vascular calcification. Kronmal et al. found that male gender was associated with the development and progression of coronary calcification[21]. We therefore included gender in the models.

**Age**: Increase in age is another long-recognized risk factor of vascular calcification. In the aforementioned study conducted by Kronmal et al., they found the annual incidence of coronary calcification increases with age[21]. We therefore included age in the models.

**Diabetes**: As demonstrated earlier, diabetes is a risk factor of vascular calcification. The aforementioned study by Kronmal et al. also found that diabetes was associated with the development and progression of coronary calcification[21]. It was therefore included in the models.

**Current use of aspirin**: This is defined as usage of aspirin within the past 7 days prior to taking CT exam. We have repetitively pointed out the correlation between atherosclerosis and intimal calcification. Aspirin is prescribed to patients with increased cardiovascular risk, including those with dyslipidemia, diabetes and established atherosclerotic cardiovascular diseases (e.g., CHD). It therefore served as a surrogate of atherosclerotic cardiovascular diseases (ASCVDs) like CHD and a marker of coexistence of cardiovascular risk factors. We noticed from clinical practice that self-reported history of CHD is not always accurate, as some patients were diagnosed of CHD without undergoing coronary computed tomography angiography or coronary digital subtraction angiography, which may be proved to be misdiagnoses. This impairs the utility of our prediction model when our model is applied to clinical settings other than that in our hospital had we directly put CHD in our models. Aspirin has been so famous and popular that according to our observation and experience in clinical practice, even patients with limited literacy know this medication and whether he or she is taking it right now. Substituting aspirin for certain names of ASCVDs or other risk factors enhances the generalizability of models by making the variables easier to understand for a broader range of populations.

**Estimated glomerular filtration rate**: Medial calcification is a hallmark of CKD[22]. CACS>100 has been found to associate with lower eGFR in a Korean population of 1,533 patients[23]. In the Rotterdam Study, association between decreased eGFR and increased CACS in the subpopulation of patients aged 70 years or older was found[24]. We therefore included eGFR in the models.

**Ratio of LDL-C and HDL-C (LDL-C/HDL-C)**: the association between LDL-C and CAC has been introduced. A cross-sectional study revealed the association of higher LDL-C/HDL-C and presence of CAC[25]. A comparative study found stronger associations between HDL-C and CACS than that of LDL-C and CACS[26].

1. **Supplemental Results**

Table S1 Number of observations with missing values on the predictors listed in Table 1.

| Predictors | No. |
| --- | --- |
| Age | 0 |
| Gender | 0 |
| Hypertension | 2 |
| Diabetes | 1 |
| Current smoking | 51 |
| Current alcohol drinking | 45 |
| Aspirin^†^ | 21 |
| Statins^†^ | 21 |
| Alkaline phosphatase, IU/L | 110 |
| Serum calcium, mmol/L | 75 |
| Serum phosphorus, mmol/L | 74 |
| Product of serum calcium and serum phosphorus, (mmol/L)^2^ | 75 |
| Serum creatinine, μmol/L | 6 |
| eGFR, mL/(min·1.73 m^2^) | 5 |
| Total cholesterol, mmol/L | 8 |
| Homocysteine, μmol/L | 133 |
| Glycated hemoglobin A1c, % | 128 |
| NT-proBNP, pg/mL | 238 |
| Lipoprotein(a), mg/L | 19 |
| Triglyceride, mmol/L | 8 |
| HDL-C, mmol/L | 8 |
| LDL-C, mmol/L | 8 |
| LDL-C/HDL-C | 8 |

eGFR indicates estimated glomerular filtration rate; NT-proBNP, N-terminal brain natriuretic peptide; No., number; HDL-C, high-density lipoprotein cholesterol; LDL-C, low-density lipoprotein cholesterol.

^†^“Aspirin” and “Statins” stand for usage of the two medications in the past 7 days prior to laboratory tests.

**Table S2 Number and proportions of missing data as well as missingness patterns in the modeling process of Models 1 and 2**

| Pattern No. | Gender | Age (years) | LDL-C | Diabetes | Current Smoking | Aspirin | Statins | No. of records with this pattern | Proportion of records with this pattern (%) |
| --- | --- | --- | --- | --- | --- | --- | --- | --- | --- |
| 1 | √ | √ | √ | √ | √ | √ | √ | 624 | 89.91 |
| 2 | √ | √ | √ | √ | √ | √ | X | 1 | 0.14 |
| 3 | √ | √ | √ | √ | √ | X | √ | 1 | 0.14 |
| 4 | √ | √ | √ | √ | √ | X | X | 12 | 1.73 |
| 5 | √ | √ | √ | √ | X | √ | √ | 41 | 5.91 |
| 6 | √ | √ | √ | √ | X | X | X | 6 | 0.86 |
| 7 | √ | √ | √ | X | X | X | X | 1 | 0.14 |
| 8 | √ | √ | X | √ | √ | √ | √ | 5 | 0.72 |
| 9 | √ | √ | X | √ | X | √ | √ | 2 | 0.29 |
| 10 | √ | √ | X | √ | X | X | X | 1 | 0.14 |

Notes: A tick in the cell represents that the corresponding variable is not missing while a cross represents the otherwise. "Aspirin" and "Statins" refer to usage of the medications. Abbreviation: LDL-C: low-density lipoprotein cholesterol, No.: number.

**Table S3 Number and proportions of missing data as well as missingness patterns in the modeling process of Model 3**

| Pattern No. | Gender | Age (years) | LDL-C | Diabetes | Current Smoking | Aspirin | Statins | eGFR | No. of subjects with this missing pattern | Proportion of subjects with this missing pattern |
| --- | --- | --- | --- | --- | --- | --- | --- | --- | --- | --- |
| 1 | √ | √ | √ | √ | √ | √ | √ | √ | 624 | 89.91 |
| 2 | √ | √ | √ | √ | √ | √ | X | √ | 1 | 0.14 |
| 3 | √ | √ | √ | √ | √ | X | √ | √ | 1 | 0.14 |
| 4 | √ | √ | √ | √ | √ | X | X | √ | 12 | 1.73 |
| 5 | √ | √ | √ | √ | X | √ | √ | √ | 41 | 5.91 |
| 6 | √ | √ | √ | √ | X | X | X | √ | 6 | 0.86 |
| 7 | √ | √ | √ | X | X | X | X | √ | 1 | 0.14 |
| 8 | √ | √ | X | √ | √ | √ | √ | √ | 2 | 0.29 |
| 9 | √ | √ | X | √ | √ | √ | √ | X | 3 | 0.43 |
| 10 | √ | √ | X | √ | X | √ | √ | √ | 1 | 0.14 |
| 11 | √ | √ | X | √ | X | √ | √ | X | 1 | 0.14 |
| 12 | √ | √ | X | √ | X | X | X | X | 1 | 0.14 |

Notes: A tick in the cell represents that the corresponding variable is not missing while a cross represents the otherwise. "Aspirin" and "Statins" refer to usage of the medications. Abbreviation: eGFR: estimated glomerular filtration rate, LDL-C: low-density lipoprotein cholesterol, No.: number.

**Table S4 Number and proportions of missing data as well as missingness patterns in the modeling process of Model 4**

| Pattern No. | Gender | Age (years) | LDL-C | HDL-C | Diabetes | Current Smoking | eGFR | No. of subjects with this missing pattern | Proportion of subjects with this missing pattern |
| --- | --- | --- | --- | --- | --- | --- | --- | --- | --- |
| 1 | √ | √ | √ | √ | √ | √ | √ | 638 | 91.93 |
| 2 | √ | √ | √ | √ | √ | X | √ | 47 | 6.77 |
| 3 | √ | √ | √ | √ | X | X | √ | 1 | 0.14 |
| 4 | √ | √ | X | X | √ | √ | √ | 2 | 0.29 |
| 5 | √ | √ | X | X | √ | √ | X | 3 | 0.43 |
| 6 | √ | √ | X | X | √ | X | √ | 1 | 0.14 |
| 7 | √ | √ | X | X | √ | X | X | 2 | 0.29 |

Notes: A tick in the cell represents that the corresponding variable is not missing while a cross represents the otherwise. Abbreviation: eGFR: estimated glomerular filtration rate, HDL-C: high-density lipoprotein cholesterol, LDL-C: low-density lipoprotein cholesterol, No.: number.

**Table S5 Number and proportions of missing data as well as missingness patterns in the modeling process of Model 5**

| Pattern No. | Gender | Age (years) | LDL-C | Diabetes | Current Smoking | eGFR | No. of subjects with this missing pattern | Proportion of subjects with this missing pattern |
| --- | --- | --- | --- | --- | --- | --- | --- | --- |
| 1 | √ | √ | √ | √ | √ | √ | 638 | 91.93 |
| 2 | √ | √ | √ | √ | X | √ | 47 | 6.77 |
| 3 | √ | √ | √ | X | X | √ | 1 | 0.14 |
| 4 | √ | √ | X | √ | √ | √ | 2 | 0.29 |
| 5 | √ | √ | X | √ | √ | X | 3 | 0.43 |
| 6 | √ | √ | X | √ | X | √ | 1 | 0.14 |
| 7 | √ | √ | X | √ | X | X | 2 | 0.29 |

Notes: A tick in the cell represents that the corresponding variable is not missing while a cross represents the otherwise. Abbreviation: eGFR: estimated glomerular filtration rate, HDL-C: high-density lipoprotein cholesterol, LDL-C: low-density lipoprotein cholesterol, No.: number.

**References:**

[1] Finn AV, Coronary Calcium: A Comprehensive Understanding of Its Biology, Use in Screening, and Interventional Management, 1 ed., Elsevier Science & Technology, Amsterdam, Netherlands, 2019.

[2] Demer LL, Tintut Y, Inflammatory, metabolic, and genetic mechanisms of vascular calcification, ARTERIOSCLEROSIS THROMBOSIS AND VASCULAR BIOLOGY, 34 (2014) 715-723.

[3] Wexler L, Brundage B, Crouse J, Detrano R, Fuster V, Maddahi J, et al., Coronary artery calcification: pathophysiology, epidemiology, imaging methods, and clinical implications. A statement for health professionals from the American Heart Association. Writing Group, CIRCULATION, 94 (1996) 1175-1192.

[4] Nakamura S, Ishibashi-Ueda H, Niizuma S, Yoshihara F, Horio T, Kawano Y, Coronary calcification in patients with chronic kidney disease and coronary artery disease, Clinical Journal of the American Society of Nephrology, 4 (2009) 1892-1900.

[5] Starkman HS, Cable G, Hala V, Hecht H, Donnelly CM, Delineation of prevalence and risk factors for early coronary artery disease by electron beam computed tomography in young adults with type 1 diabetes, DIABETES CARE, 26 (2003) 433-436.

[6] Kamenskiy A, Poulson W, Sim S, Reilly A, Luo J, MacTaggart J, Prevalence of Calcification in Human Femoropopliteal Arteries and its Association with Demographics, Risk Factors, and Arterial Stiffness, ARTERIOSCLEROSIS THROMBOSIS AND VASCULAR BIOLOGY, 38 (2018) e48-e57.

[7] Janssen T, Bannas P, Herrmann J, Veldhoen S, Busch JD, Treszl A, et al., Association of linear ¹⁸F-sodium fluoride accumulation in femoral arteries as a measure of diffuse calcification with cardiovascular risk factors: a PET/CT study, JOURNAL OF NUCLEAR CARDIOLOGY, 20 (2013) 569-577.

[8] Derlin T, Tóth Z, Papp L, Wisotzki C, Apostolova I, Habermann CR, et al., Correlation of inflammation assessed by 18F-FDG PET, active mineral deposition assessed by 18F-fluoride PET, and vascular calcification in atherosclerotic plaque: a dual-tracer PET/CT study, JOURNAL OF NUCLEAR MEDICINE, 52 (2011) 1020-1027.

[9] Castagna MT, Mintz GS, Ohlmann P, Kotani J, Maehara A, Gevorkian N, et al., Incidence, location, magnitude, and clinical correlates of saphenous vein graft calcification: an intravascular ultrasound and angiographic study, CIRCULATION, 111 (2005) 1148-1152.

[10] Jadidi M, Poulson W, Aylward P, MacTaggart J, Sanderfer C, Marmie B, et al., Calcification prevalence in different vascular zones and its association with demographics, risk factors, and morphometry, AMERICAN JOURNAL OF PHYSIOLOGY-HEART AND CIRCULATORY PHYSIOLOGY, 320 (2021) H2313-H2323.

[11] Hisamatsu T, Miura K, Arima H, Kadota A, Kadowaki S, Torii S, et al., Smoking, Smoking Cessation, and Measures of Subclinical Atherosclerosis in Multiple Vascular Beds in Japanese Men, Journal of the American Heart Association, 5 (2016) e003738.

[12] Libby P, Inflammation in atherosclerosis, ARTERIOSCLEROSIS THROMBOSIS AND VASCULAR BIOLOGY, 32 (2012) 2045-2051.

[13] Henein M, Cardiovascular Calcification, 1 ed., Springer, Cham, Switzerland, 2021.

[14] Hao QY, Gao JW, Yuan ZM, Gao M, Wang JF, Schiele F, et al., Remnant Cholesterol and the Risk of Coronary Artery Calcium Progression: Insights From the CARDIA and MESA Study, Circulation-Cardiovascular Imaging, 15 (2022) e014116.

[15] Mortensen MB, Falk E, Li D, Nasir K, Blaha MJ, Sandfort V, et al., Statin Trials, Cardiovascular Events, and Coronary Artery Calcification: Implications for a Trial-Based Approach to Statin Therapy in MESA, JACC-Cardiovascular Imaging, 11 (2018) 221-230.

[16] Hong SP, Kim CY, Jung HW, The Comparison of the Associations of Lipoprotein(a) and the Atherogenic Index of Plasma With Coronary Artery Calcification in Patients Without High LDL-C: A Comparative Analysis, J Lipid Atheroscler, 12 (2023) 152-163.

[17] Schmermund A, Achenbach S, Budde T, Buziashvili Y, Förster A, Friedrich G, et al., Effect of intensive versus standard lipid-lowering treatment with atorvastatin on the progression of calcified coronary atherosclerosis over 12 months: a multicenter, randomized, double-blind trial, CIRCULATION, 113 (2006) 427-437.

[18] Hozawa A, Folsom AR, Sharrett AR, Payne TJ, Chambless LE, Does the impact of smoking on coronary heart disease differ by low-density lipoprotein cholesterol level?: the Atherosclerosis Risk in Communities (ARIC) Study, CIRCULATION JOURNAL, 70 (2006) 1105-1110.

[19] Nakazato R, Gransar H, Berman DS, Cheng VY, Lin FY, Achenbach S, et al., Statins use and coronary artery plaque composition: results from the International Multicenter CONFIRM Registry, ATHEROSCLEROSIS, 225 (2012) 148-153.

[20] Puri R, Nicholls SJ, Shao M, Kataoka Y, Uno K, Kapadia SR, et al., Impact of statins on serial coronary calcification during atheroma progression and regression, JOURNAL OF THE AMERICAN COLLEGE OF CARDIOLOGY, 65 (2015) 1273-1282.

[21] Kronmal RA, McClelland RL, Detrano R, Shea S, Lima JA, Cushman M, et al., Risk factors for the progression of coronary artery calcification in asymptomatic subjects: results from the Multi-Ethnic Study of Atherosclerosis (MESA), CIRCULATION, 115 (2007) 2722-2730.

[22] Shao JS, Cai J, Towler DA, Molecular mechanisms of vascular calcification: lessons learned from the aorta, ARTERIOSCLEROSIS THROMBOSIS AND VASCULAR BIOLOGY, 26 (2006) 1423-1430.

[23] Hyun YY, Kim H, Oh KH, Ahn C, Park SK, Chae DW, et al., eGFR and coronary artery calcification in chronic kidney disease, EUROPEAN JOURNAL OF CLINICAL INVESTIGATION, 49 (2019) e13101.

[24] El BA, Elias-Smale S, Dehghan A, Vliegenthart-Proença R, Oudkerk M, Hofman A, et al., Renal function is related to severity of coronary artery calcification in elderly persons: the Rotterdam study, PLoS One, 6 (2011) e16738.

[25] Hisamatsu T, Fujiyoshi A, Miura K, Ohkubo T, Kadota A, Kadowaki S, et al., Lipoprotein particle profiles compared with standard lipids in association with coronary artery calcification in the general Japanese population, ATHEROSCLEROSIS, 236 (2014) 237-243.

[26] Allison MA, Wright CM, A comparison of HDL and LDL cholesterol for prevalent coronary calcification, INTERNATIONAL JOURNAL OF CARDIOLOGY, 95 (2004) 55-60.
